# Editorial: Novel approaches to diet, exercise, and drugs in childhood obesity and metabolic diseases

**DOI:** 10.3389/fped.2025.1685450

**Published:** 2025-09-04

**Authors:** Kexin Zhang, Xuan Li, Sen Li, Xiaodong Sun

**Affiliations:** ^1^ Department of Endocrinology and Metabolism, Shandong Provincial Key Medical and Health Laboratory of Endocrinology and Metabolic Diseases, Affiliated Hospital of Shandong Second Medical University, Weifang, China; ^2^ Clinical Research Center, Affiliated Hospital of Shandong Second Medical University, Weifang, China; ^3^ Department of Physiology and Biophysics, University of Mississippi Medical Center, Jackson, MS, United States; ^4^ School of Life Sciences, Beijing University of Chinese Medicine, Beijing, China

**Keywords:** childhood obesity, metabolic diseases, dietary interventions, physical activity, drug

**Editorial on the Research Topic**
Novel approaches to diet, exercise, and drugs in childhood obesity and metabolic diseases

Childhood obesity and related metabolic disorders represent one of the most pressing public health challenges of the 21st century (1, 2). Globally, prevalence continues to rise, with obesity appearing at increasingly younger ages and bringing earlier onset of metabolic dysregulation, elevated cardiovascular risk, and psychosocial consequences (3, 4). Although body mass index (BMI) remains the most widely used diagnostic tool, it fails to fully capture the complex nature of obesity, particularly in diverse pediatric populations with varying body compositions, activity levels, and socio-environmental exposures (5). Emerging research highlights the importance of early-life behavioral, environmental, and metabolic determinants, many of which are modifiable if addressed promptly (6, 7). This research topic brings together five original research articles and two study protocols that collectively showcase advances in understanding, preventing, and managing pediatric obesity and its cardiometabolic problems.

Growing evidence highlights the importance of early, family-centered approaches as a foundation for obesity prevention (8). The Feeding the Family—The Intergenerational Approach to Fight Obesity (FACILITY) study by Vincenti et al. presents a timely and innovative model that explores how maternal, child, socioeconomic, cultural, and environmental factors interact to influence obesity risk. By focusing on mother–child pairs and capturing a broad range of variables, the study offers a robust framework for primordial prevention. The findings have the potential to guide tailored public health strategies, particularly for vulnerable populations. Despite its single-center design, the study's intergenerational approach shows strong potential for shaping equitable and culturally sensitive obesity prevention policies.

School-based interventions represent a valuable opportunity to promote healthy behaviors early in life. Dong et al. illustrate this approach through the Wuhan Preschool Healthy Start project, an innovative cluster-randomized trial that incorporates self-regulation strategies into preschoolers' daily routines. The program focuses on improving diet, physical activity, sleep, and reducing sedentary behavior, while also strengthening both general and food-related self-regulation. It actively involves children, their families, and schools. By encouraging collaboration between home and school and promoting healthy habits in all areas of a child's life, the program aims to support lasting behavior change and healthier growth patterns. If successful, the results could guide national and international public health strategies, highlighting self-regulation as a key element in preventing obesity from an early age.

Refining obesity assessment tools is critical to prevent misclassification and guide appropriate interventions. Ayuzo Del Valle et al. introduced the 2025 Obesity Classification Framework, which integrates body composition, waist-to-height ratio, and metabolic markers for a more individualized evaluation. Among young athletes, this method distinguishes excess adiposity from lean mass, reducing unnecessary interventions and enabling earlier identification of preclinical obesity. While this risk-centered paradigm holds the potential to transform pediatric obesity care, further validation across diverse populations and consideration of psychosocial implications will be critical for widespread adoption.

Behavioral factors, particularly eating patterns, play a decisive role in childhood fat distribution. Wang et al. demonstrate through cross-sectional and prospective analyses that food responsiveness, emotional overeating, and emotional undereating are consistently linked to greater visceral and trunk fat mass. These findings highlight the importance of integrating behavioral, nutritional, and psychological components into prevention programs. Addressing maladaptive eating behaviors during primary school years could significantly reduce the long-term metabolic risks associated with abdominal obesity.

Regular physical activity plays a vital role in safeguarding the mental well-being of children and adolescents, especially in the face of rising overweight and obesity rates. The study by Andrade et al. demonstrates that active youth report lower levels of depression, anger, fatigue, and confusion, alongside greater vigor, than their inactive peers. Importantly, higher BMI was linked to more negative mood states, underscoring the intertwined nature of physical and emotional health. These findings emphasize the need for integrated interventions that combine physical activity, weight management, and mental health support to build healthier and more resilient youth.

Endocrine and developmental disorders can intersect with metabolic dysregulation in complex ways. Childhood central precocious puberty (CPP) is increasingly recognized as both a metabolic and developmental concern. Cui et al. report that children with CPP have higher fasting glucose, hemoglobin A1c, triglycerides, total cholesterol, and low-density lipoprotein cholesterol levels, alongside lower high-density lipoprotein cholesterol, compared with peers without CPP. Obesity was more prevalent in the CPP group, and Tanner stage showed a positive correlation with BMI. These results underscore the need for early metabolic screening and weight management in children with CPP to mitigate their heightened risk for future cardiovascular and endocrine diseases.

Childhood cardiovascular disease is an emerging consequence of the global obesity epidemic. Using Global Burden of Disease data, Wang et al. reveal a 25% increase in global cardiovascular disease incidence among children from 1990 to 2021, particularly among older children. Although mortality and disability-adjusted life years have declined, low socio-demographic index regions bear a disproportionate burden due to obesity, diabetes, environmental exposures, and healthcare inequities. These findings call for context-specific strategies that combine medical prevention with socio-economic improvements, ensuring equitable access to care and healthier futures for children worldwide.

In conclusion, these studies show that tackling childhood obesity requires early, integrated, and context-specific strategies spanning family, school, and community settings. Precise assessment, behavioral and psychological support, and attention to comorbidities are essential, along with efforts to address global health disparities. Coordinated action can help curb the obesity epidemic and secure healthier futures for children worldwide.
